# Associations of Haplotypes Upstream of *IRS1* with Insulin Resistance, Type 2 Diabetes, Dyslipidemia, Preclinical Atherosclerosis, and Skeletal Muscle *LOC646736* mRNA Levels

**DOI:** 10.1155/2015/405371

**Published:** 2015-05-18

**Authors:** Selma M. Soyal, Thomas Felder, Simon Auer, Hannes Oberkofler, Bernhard Iglseder, Bernhard Paulweber, Silvia Dossena, Charity Nofziger, Markus Paulmichl, Harald Esterbauer, Franz Krempler, Wolfgang Patsch

**Affiliations:** ^1^Institute of Pharmacology and Toxicology, Paracelsus Medical University, 5020 Salzburg, Austria; ^2^Department of Laboratory Medicine, Paracelsus Medical University, 5020 Salzburg, Austria; ^3^Department of Geriatric Medicine, Paracelsus Medical University, 5020 Salzburg, Austria; ^4^Department of Internal Medicine I, Paracelsus Medical University, 5020 Salzburg, Austria; ^5^Department of Laboratory Medicine, Medical University of Vienna, 1090 Vienna, Austria; ^6^Department of Internal Medicine, Krankenhaus Hallein, 5400 Hallein, Austria

## Abstract

The genomic region ~500 kb upstream of *IRS1* has been implicated in insulin resistance, type 2 diabetes, adverse lipid profile, and cardiovascular risk. To gain further insight into this chromosomal region, we typed four SNPs in a cross-sectional cohort and subjects with type 2 diabetes recruited from the same geographic region. From 16 possible haplotypes, 6 haplotypes with frequencies >0.01 were observed. We identified one haplotype that was protective against insulin resistance (determined by HOMA-IR and fasting plasma insulin levels), type 2 diabetes, an adverse lipid profile, increased C-reactive protein, and asymptomatic atherosclerotic disease (assessed by intima media thickness of the common carotid arteries). BMI and total adipose tissue mass as well as visceral and subcutaneous adipose tissue mass did not differ between the reference and protective haplotypes. In 92 subjects, we observed an association of the protective haplotype with higher skeletal muscle mRNA levels of *LOC646736*, which is located in the same haplotype block as the informative SNPs and is mainly expressed in skeletal muscle, but only at very low levels in liver or adipose tissues. These data suggest a role for *LOC646736* in human insulin resistance and warrant further studies on the functional effects of this locus.

## 1. Introduction

Insulin resistance (IR) and impaired upregulation of insulin secretion are hallmarks of type 2 diabetes. IR is thought to be central to the metabolic syndrome [1] and, even in the absence of type 2 diabetes, it enhances the risk of cardiovascular disease [2]. While IR is strongly associated with obesity, it also occurs in lean individuals [1]. Genome-wide associations studies (GWAS) implicated several loci in IR pathways [3, 4]. rs2943641, located ~500 kb upstream of* IRS1*, was the first type 2 diabetes risk locus identified in a GWAS that was linked to IR and hyperinsulinemia [5]. In addition, rs2943641 or SNPs in strong linkage disequilibrium (LD) with it displayed associations with coronary artery disease [6] and HDL cholesterol and/or triglycerides (TG) in other populations [7, 8], but not with BMI. To gain further insight into the role of this chromosomal region in IR and related phenotypes, we typed three additional SNPs in the LD block harboring rs2943641 in a cross-sectional cohort and in subjects with type 2 diabetes, all recruited from the same geographical region. We describe here a common protective haplotype against type 2 diabetes that is associated with reduced levels of surrogate IR markers and average intima media thickness (IMT) of the common carotid arteries, but not obesity or indices of fat mass or distribution. As* IRS1* is located >500 kb downstream of the genomic region harboring the informative SNPs and the size of associations is typically less than 200 kb [9], we performed an initial characterization of* LOC646736*, which is located within the same LD block as the informative SNPs.

## 2. Materials and Methods

### 2.1. Study Populations

The two study populations comprised white Europeans, living in the same geographic region. SNPs and haplotype frequencies were analyzed in 1028 male, 40 to 65 years old, and 622 female, 45 to 70 years old, participants of the Salzburg Atherosclerosis Prevention Program in Subjects of High Individual Risk (SAPHIR). Study design and recruitment procedures have been described [10] and clinical data were obtained between 1999 and 2003. Associations of SNPs and haplotypes with anthropometric and metabolic parameters were ascertained in 983 male and 599 female SAPHIR participants without type 2 diabetes. Diabetes was diagnosed by fasting plasma glucose concentrations of ≥126 mg/dL and/or use of hypoglycemic medications. Associations with type 2 diabetes were determined in 600 patients with type 2 diabetes and 1350 SAPHIR participants who were not using hypoglycemic medications and had fasting glucose levels <100 mg/dL and 2 h OGTT values <140 mg/dL. Type 2 diabetes cases included 68 SAPHIR participants and 532 unrelated patients, recruited from the outpatient clinics of the University Hospital Salzburg and the Krankenhaus Hallein between 1999 and 2002. Clinical data of the populations used for these comparisons are shown in Supplementary Tables 1–3 (in Supplementary Material available online at http://dx.doi.org/10.1155/2015/405371). Biopsies from the rectus abdominis muscle, visceral and subcutaneous adipose tissues (VAT and SAT, resp.), and liver of obese subjects were obtained during bariatric surgery between 1998 and 2005. Such tissue samples were also obtained from lean control subjects undergoing an elective surgical procedure such as repair of hernias and cholecystectomy during the same time period [11–13]. Tissue samples were placed immediately in RNA-later (Qiagen) and stored at −80°C. The study protocols were approved by the local ethics committee. Informed consent was obtained from all patients included in the study.

Fat and lean body mass were determined in SAPHIR participants by bioelectric impedance analysis using the phase-sensitive, fully digital multifrequency analyzer BIA-2000M (Data Input, Hofheim, Germany). Abdominal adipose tissue areas were assessed by computed tomography using MX TWIN Picker CT scanner (Marconi Medical Systems, Cleveland, OH) in 897 men and 540 women without type 2 diabetes as described in [14]. An abdominal scan was obtained between the fourth and fifth lumbar vertebrae (L4-L5), and the adipose tissue area was determined by calculating the pixel distribution with attenuation values between −190 and −30 Hounsfield units. The abdominal visceral fat area was determined by drawing a line within the muscle wall surrounding the abdominal cavity. The encircled area with attenuation values between −190 and −30 Hounsfield units was then calculated using ht Voxel Q software package (Marconi Medical Systems). Abdominal subcutaneous fat was determined by subtracting visceral from abdominal total fat.

Intima media thickness (IMT) of the near and far walls of the common arteries and the bifurcations on both sides were measured by high resolution B-mode ultrasound using the HDI 3000 CV System (ATL, Munich, Germany) according to the ACAPS protocol as described in [15]. All measurements were conducted and read by a single experienced ultrasound operator who was blinded to all clinical and laboratory measurements. In short, the scanning protocol included multiple longitudinal and transverse imaging planes of the common carotid artery 8 mm proximal to the bifurcation, at the height of the flow divider. Intima media thickness was measured at end diastole as the distance from the leading edge of the second bright line of the far wall (lumen-intima interface) to the leading edge of the second bright line (media-adventitia interface); for the near wall, the best estimate of the IMT is the distance between the trailing edge of the first bright line and the trailing edge of the second bright line. Values used for statistical analyses were defined as the average of near and far wall measurements of both the right and the left common carotid arteries. Thus, average IMTs represent the mean from all individual measurements. Study participants with symptomatic carotid artery disease were excluded. Due to technical difficulties, complete IMT measurements were available in 943 men and 599 women. Hypertension was defined by systolic and/or diastolic blood pressure readings of >140 and/or 90 mg Hg or use of antihypertensive drugs. Clinical data of subjects used in these analyses are shown in Supplementary Table 4.

### 2.2. Laboratory Determination

Venous blood was drawn into EDTA-containing tubes after an overnight fast. Laboratory parameters were determined as described in [13].

### 2.3. Genotyping

DNA was isolated from the whole blood cells using the Generation Capture Column Kit (Gentra Systems) and stored at −20°C. We genotyped rs7578326, rs2943634, rs2943641, and rs2713538 using TaqMan Genotyping Assays (Applied Biosystems) and C_29270128_10, C_15949679_10, C_1533178_10, and C_1533153_10, respectively. The accuracy of genotyping was verified by sequencing in 30 subjects.

### 2.4. Myoblast Isolation and Cell Cultures

Primary human skeletal muscle cells were isolated, cultured, and differentiated by established methods [16, 17] as described in [18]. In brief, satellite cells from human rectus abdominis muscle biopsies were released from myotubes by enzymatic digestion. Contaminating fibroblasts were removed by adhesion of myoblasts to CD56 (Leu 19; Becton Dickinson, Franklin Lakes, MJ, USA), which was immobilized onto magnetic beads (CELLection Pan Mouse IgG Kit, Life Technologies). The purified myoblast population was plated on collagen-coated dishes and expanded in skeletal muscle cell growth medium (SkGM Bullet Kit, Lonza, Basel, Switzerland). SH-SY5Y cells were obtained from ATCC and cultured in DMEM/F12 1 : 1 (Invitrogen). C2C12 cells were cultured in DMEM. Culture media were supplemented with 10% fetal bovine serum and 0.01% penicillin/streptomycin.

### 2.5. RNA Sources

Total RNA was isolated from human skeletal muscle, VAT, SAT, and liver biopsies using the RNeasy Lipid Tissue Midi Kit or the RNeasy Kit (Qiagen). A human multiple tissue panel was purchased from Ambion.

### 2.6. Transcript Cloning and Plasmids

DNase I-treated total human skeletal muscle RNA (1 *μ*g/reaction) was reverse transcribed as described in [19]. cDNAs were amplified by PCR using primers located in exon 1 and exon 7 of the* LOC646736* reference gene. PCR products were subcloned into the PGEM T-Easy vector (Promega). Plasmids for* in vitro* transcription/translation included the coding sequences of* LOC646736*_ex1-3 and* LOC646736*_ex5-7 transcripts cloned into the* Bam*HI/*Xho*I sites of the pT7CFE1-CHis expression vector (Thermo Scientific). For transfection studies, the same coding sequences were cloned into the* Xho*I/*Bam*HI sites of the pEGFP-N1 vector (Clontech). Respective primers are listed in Supplementary Tables 5–7. Sequences of all constructs were verified using the BigDye Terminator v3.1 Cycle Sequencing Kit (Applied Biosystems) and the ABI 3500 genetic analyzer (Applied Biosystems).

### 2.7. Quantitative RT-PCR Analysis

RNA from rectus abdominis skeletal muscle of 73 obese (23 males and 50 females) and 19 lean controls (9 males and 10 females) was extracted as described in [19]. Participants were included if they had no type 2 diabetes, no history of use of lipid lowering drugs, and CRP plasma levels <20 mg/L. Clinical data of these subjects are shown in Supplementary Table 8. RNA was also extracted from liver and adipose tissues of 1 male and three female subjects fulfilling the criteria described above. RNA was reverse transcribed and cDNAs were subjected to real-time PCR using the iQ SYBR Green Supermix (Bio-Rad) and primers targeting exon 6 and exon 7 of* LOC646736* (Supplementary Table 5).* IRS1* transcripts were quantified by real-time PCR using primers described in [20] (Supplementary Table 5). The absence of genomic DNA was verified by performing amplification of 1 *μ*g RNA without prior reverse transcription *C*_*T*_ values for* LOC646736* and* IRS1* transcripts were normalized for the expression of* RPLP0* as described in [18].

### 2.8. *In Vitro* Transcription/Translation and Immunoblotting

The 1-step Human Coupled IVT Kit-DNA (Thermo Scientific) was used for* in vitro* synthesis of* LOC646736* proteins [19]. Circular plasmids (1 *μ*g) pCFE-*LOC646736*_ex1-3 and pCFE-*LOC646736*_ex5-7 were each incubated with 12.5 *μ*L HeLa Lysate, 2.5 *μ*L accessory proteins, and 5 *μ*L reaction mix in a total volume of 25 *μ*L for 90 min at 30°C. Samples were denatured in sample buffer (62.5 mM Tris-HCl, pH 6.8, 2% w/v SDS, 10% glycerol, 50 mM dithiothreitol, and 0.01% bromophenol blue) at 70°C for 10 min, cooled on ice, and subjected to electrophoresis in 12% SDS-polyacrylamide gels and transferred to polyvinylidene fluoride (PVDF) membranes (GE Healthcare, Life Sciences, Buckinghamshire, England). Membranes were blocked with 5% nonfat dry milk diluted in wash buffer (0.1% Tween-20 in Tris-buffered saline, pH 7.6) for 2 h at room temperature, incubated with THE His Tag Antibody (GenScript, Piscataway, NJ, USA) overnight at 4°C, washed 5 times, and incubated with the secondary antibody, IRDye 800CW goat anti-mouse IgG (Li-cor, Superior St. Lincoln, NE, USA) for 1 h at room temperature. After three 5-min incubations with wash buffer, proteins were visualized using the Odyssey infrared imaging system (Li-COR).

### 2.9. Confocal Microscopy

Primary human myoblasts, C2C12 cells and SH-SY5Y cells were plated on cover slips in six-well dishes and transfected with 500 ng of plasmids pLOC646736_ex1-3_GFP or pLOC646736_ex5-7_GFP for 24 h. Cells were stained with 100 nM MitoTracker Red (Invitrogen) for 45 min, rinsed three times for 15 min with PBS, fixed with 4% paraformaldehyde, rinsed with PBS, and stained with DAPI (4′,6′-diamidino-2-phenylindole, Sigma-Aldrich) for 30 min. Slides were mounted with DAPCO and Mowiol (Sigma-Aldrich). A Zeiss LSM710 confocal microscope equipped with an Axiocam digital camera and an oil-immersion ×63 objective lens was used for imaging [19].

### 2.10. Statistics

Allele frequencies were estimated by gene counting. Agreement with Hardy-Weinberg expectations was tested using a *χ* ^2^ goodness-of-fit test. The standardized pairwise linkage disequilibrium statistics (*D*′) and haplotype frequencies were estimated and effects of genotypes (associated with individual SNPs) on continuous variables were ascertained by ANOVA. Logarithmic transformations were made if the equal variance and normality assumptions of ANOVA were rejected. For associations of* LOC646736* and* IRS1* transcript levels with SNPs and haplotypes, log-transformed transcript levels were used. Differences in genotype frequencies between controls and patients with T2DM were calculated using a *χ* ^2^ distribution. To estimate odds ratios (ORs) with confidence intervals (95% CI) for each genotype, two “dummy” variables with the respective wild-type as the reference were used in univariate logistic regression analysis. Adjustments were made by including covariates in a second set of multivariate logistic regression models. The THESIAS program (http://genecanvas.ecgene.net) was used for testing associations between haplotypes and phenotypes. Covariate-adjusted haplotype-phenotype parameters, expressed as OR for binary phenotypes or average effects for continuous variables, were estimated for each haplotype by comparison to the most frequent haplotype.

## 3. Results

Genotypes determined in 1650 SAPHIR participants fulfilled Hardy-Weinberg expectations and did not differ by sex. *D*′ and *r* ^2^ values ranged from −0.75 to 0.92 and 0.431 to 0.796, respectively (Supplementary Tables 9 and 10). Out of the 16 possible haplotypes, six haplotypes with an estimated frequency >0.01 were observed (Table 1). For each of the common haplotypes, the squared correlation between true and predicted haplotype dose was >0.91. In 1582 SAPHIR participants without type 2 diabetes, we observed nominal associations of rs7578326 with fasting insulin levels and HOMA-IR (*P* < 0.05). rs2943634 showed borderline associations with TG (*P* = 0.05) and HDL cholesterol (*P* = 0.06) and rs2713538 with fasting insulin, HOMA-IR, and CRP (all *P* < 0.05, data not shown). Haplotype analysis adjusted for age, sex, and BMI displayed a global haplotype effect for HOMA-IR. In comparison to the most common haplotype 1112, fasting insulin levels and HOMA-IR values of haplotypes 1221 and 2221 were significantly higher and lower, respectively (Table 1), and the difference between 1221 and 2221 was highly significant (*P* = 0.00006). Haplotype 2221 displayed lower plasma TG levels (*P* < 0.05). However, no associations with BMI were noted. We observed a reduced VAT/SAT ratio for haplotype 1111, but no differences in body composition indices between the reference haplotype and the haplotypes with lower or higher HOMA-IR values. Interestingly, haplotypes 1111 and 2221 showed reduced CRP levels. In men, similar associations were observed as in the entire population. In women, HOMA-IR and TG for haplotype 2221 showed insignificant trends in similar directions as in males (Supplementary Tables 11 and 12).

Comparison of genotype frequencies between 600 type 2 diabetes patients and 1350 glucose tolerant SAPHIR participants revealed nominally significant effects of rs7578326, rs2943634, and rs2943641 as well as a borderline effect of rs2713538 (Supplementary Table 13). Similar associations were observed in men, but no significant associations were found in women (data not shown). Haplotype analysis in the entire population (both unadjusted and adjusted) showed a global effect and a significant risk reduction for haplotype 2221 in comparison to the reference haplotype (Table 2). Sex-specific analyses revealed similar risk reductions in men, but only insignificant trends in women. However, no heterogeneity across sex in the case-control association study was noted (Supplementary Tables 14 and 15).

We next ascertained possible associations of haplotypes with IMT measurements of the common carotid arteries, a surrogate marker for atherosclerotic disease. We adjusted the analysis for established risk factors and noted a significant difference between the reference and the 2221 haplotype shown to be associated with reduced risk of IR and type 2 diabetes (Table 3). Again, an association was observed in men, but not in women (Supplementary Tables 16 and 17).

**Figure 1:**
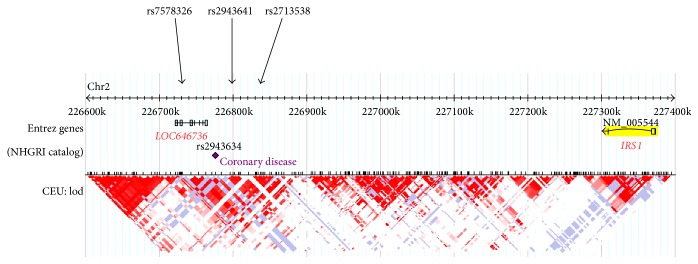
Location of typed SNPs relative to* IRS1* and* LOC646736*. Haplotype blocks are shown in the lower half of the figure (HapMap Data Phase III/Rel #2, February 2009, on NCBI B36 assembly, dbSNP b126, CEU population).

**Figure 2:**
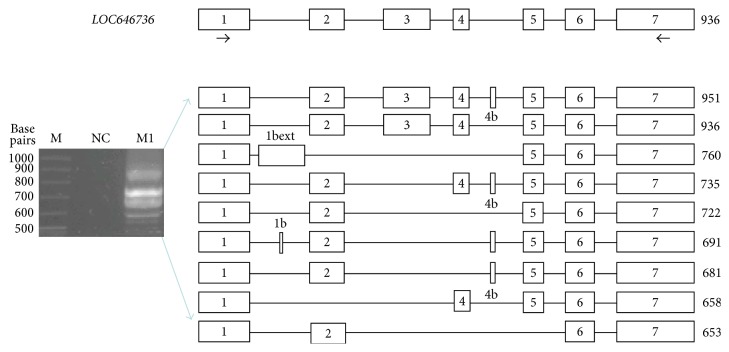
*LOC646736* structure and PCR amplification products using primers targeting exons 1 and 7 and cDNA prepared from skeletal muscle polyA^+^ RNA. Transcript structures obtained by sequencing of PCR products; M, DNA size markers; NC, negative control; and M1, human skeletal muscle.

**Figure 3:**
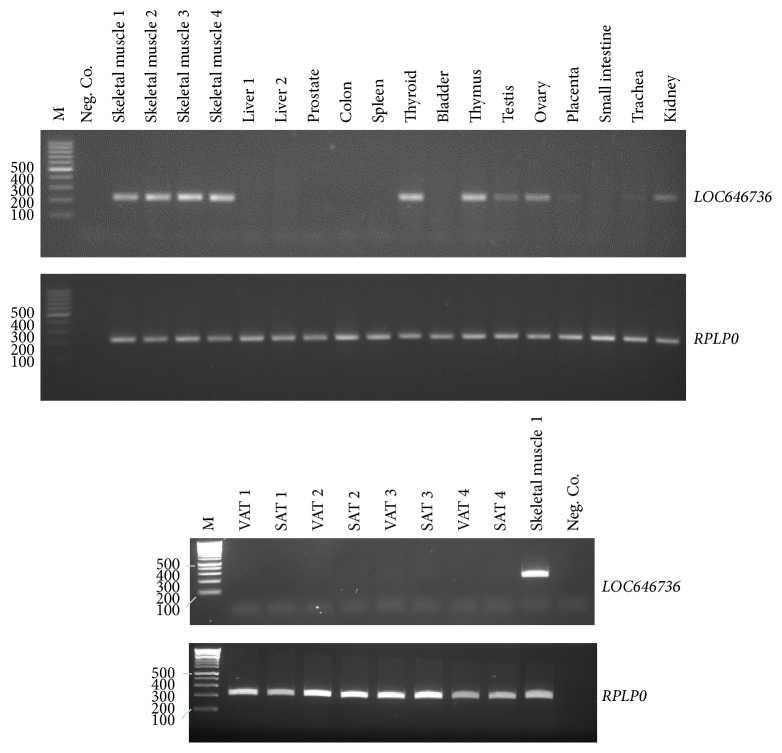
Tissue-specific expression of* LOC646736* transcripts using cDNA obtained from tissues indicated on top and primers targeting exons 6 and 7 and from visceral (VAT) or subcutaneous (SAT) adipose tissues of four obese subjects in comparison to skeletal muscle.* RPLP0* transcript abundance in the respective tissues is shown below for comparison. M, DNA marker; Neg. Co., negative control.

Several LD blocks are interspersed between* IRS1* and the region harboring the SNPs typed in this study (Figure 1). Therefore, we searched the UCSC database [21] for ESTs in the informative LD block and found four entries. They were expressed in skeletal muscle (BC017935, BF693624), fetal liver (BX646932), and a bladder carcinoma cell line (BC014369). The first three ESTs aligned with* LOC646736* comprising 7 exons and predicting a transcript of 951 nt. The start of exon 1 coincided with a highly predicted transcription start site. DNase I hypersensitive sites, hallmarks of regulatory DNA regions, have been mapped to the upstream region of exon 1, and H3K27Ac marks, found near active regulatory elements, have been identified more distally [22]. The fourth EST (BC014369) has been classified as a long intergenic noncoding RNA (lincRNA) that may be coexpressed with adjacent genes [23]. As the putative promoter contained perfect target sequences for transcription factors known to be active in skeletal muscle such as MyoD, Myf3, PEA3, HOXD8, and SBF1 [24], we performed RT-PCR in skeletal muscle samples using primers targeting exon 1 and exon 7. Electrophoretic separation of products and sequencing identified three new exons and a total of nine transcripts resulting from alternative splicing (Figure 2). As exons 6 and 7 were present in all transcripts identified, we used primers spanning these exons to determine tissue-specific expression. Reverse transcription PCR showed that* LOC646736* mRNA is mainly expressed in skeletal muscle, thyroid, and thymus and, to a lesser extent, in testes, ovary, and kidney. No amplification products were observed in liver and VAT or SAT cDNAs, indicating no or very low levels of mRNA expression (Figure 3). In skeletal muscles of 92 subjects, quantitative real-time PCR showed no difference in* LOC646736* between obese and lean subjects (Supplementary Table 8). However, significantly higher mRNA levels were associated with the protective 2221 haplotype in comparison to the reference haplotype in the whole study group. Global haplotype statistics were significant as well (Table 4). In the model considering effects of haplotypes, we noted significant sex-specific differences with higher* LOC646736* transcript levels in women (*P* = 0.006), but no significant effect of BMI (data not shown). We nevertheless analyzed haplotype effects only in obese subjects and noted a significant difference (2.77 [95% CI 0.62–4.9] arbitrary units, *P* = 0.011) between the reference and the protective haplotype. Such analyses in the smaller group of nonobese subjects revealed a similar but insignificant trend.

*IRS1* transcripts measured in skeletal muscles of the same subjects showed a higher abundance level in females than in males (*P* = 0.401), a trend towards lower levels with age (*P* = 0.08), and an association with* LOC646736* transcripts (*R* = 0.2644; *P* = 0.011). Even though the protective 2221 haplotype showed the highest expected mean* IRS1* transcript level, comparison with the reference haplotype showed no significant difference (Table 4). Among the SNPs typed, mean (SD) log-transformed* IRS1* transcript levels, adjusted for age, sex, and BMI, tended to be higher in subjects homozygous for the less common rs2943641 allele in comparison to carriers of the more common allele [1.13 (0.43) versus 0.95 (0.29), *P* = 0.059]. Furthermore, subjects homozygous for the more common allele of rs2713538 displayed nominal higher expected mean log-transformed* IRS1* transcript levels in comparison to carriers of the less common allele [1.12 (0.42) versus 0.95 (0.28), *P* = 0.034].

**Figure 4:**
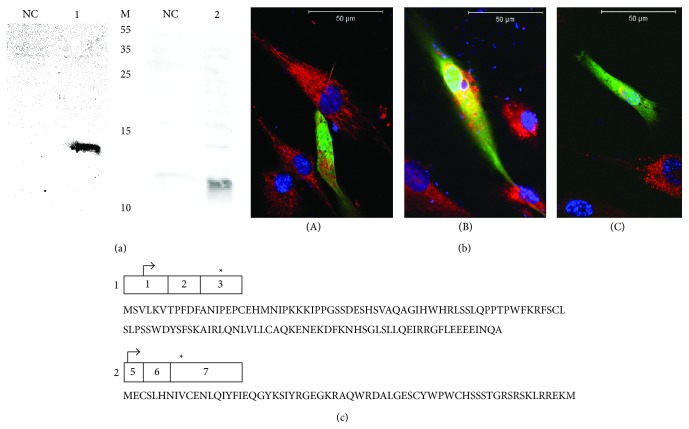
Western blots of* in vitro* translated* LOC646736_ex1*-*3* (1) and* LOC646736_ex5*-*7* (2) transcripts (a), confocal images of primary human myoblasts transfected with plasmids encoding eGFP (A),* LOC646736_ex1*-*3* (B), and* LOC646736_ex5*-*7* (C) transcripts in-frame with eGFP (b), and amino acid sequences encoded by* LOC646736_ex1*-*3* and* LOC646736_ex5*-*7* transcripts (c). NC, negative control; M, molecular weights in kDa; in B, cells were stained with MitoTracker and DAPI; in C, ∗ indicates stop codons.

*In silico* analyses of* LOC646736* transcripts showed that exon 2 has been exapted from a primate-specific Alu short interspersed element (SINE). The exonization of such elements is a mechanism of genomic diversification that appears to be specific for higher mammals [25]. Homologues of* LOC646736* were observed in greater and lesser hominoids, but not in other mammals. Our* in silico* analyses identified several open reading frames (ORFs). The largest ORF extending from exon 1 to exon 3 predicts a protein of 124 amino acids with a molecular weight of 14151 Da. Another ORF, present in several transcripts and encoded by exons 5 to 7, predicts a shorter protein of 73 amino acids with a molecular weight of 8705 Da. Clones harboring the respective coding sequences were translated in HeLa cell lysates and gave rise to proteins of the expected sizes. Furthermore, transfections of primary human myoblasts with the two sequences cloned in-frame into enhanced green fluorescent protein (eGFP) expression vectors resulted in a fluorescent signal indicating expression of the respective fusion proteins (Figures 4(a) and 4(b)). Transfections of SH-SY5Y cells and C2C12 cells displayed similar results (not shown).

## 4. Discussion

The identification of DNA variants underlying diabetes susceptibility and/or its clinical hallmarks should provide a pathophysiological perspective of disease subtypes and therefore may afford the development of more specific therapies [3, 26]. However, for many SNPs associated with type 2 diabetes in GWAS, the annotation of the gene(s) or their functional role is inconclusive. We report here a haplotype in the intergenic region between* IRS1* and the gene encoding neuronal tyrosine-phosphorylated phosphoinositide-3-kinase adapter 2 (*NYAP*,* KIAA1486*) that is protective against IR, type 2 diabetes, and preclinical atherosclerosis.

In the original study, rs2943641 was associated with several markers of IR in three populations [5]. However, no direct effect of rs2943641 on BMI was noted in normoglycemic individuals or type 2 diabetes cases. Our results confirm these studies by identifying a protective haplotype (harboring the rs2943641 minor allele) against IR and type 2 diabetes. Like in the original study, we observed no effect of this common haplotype on BMI in a cross-sectional study. In line with other studies [7, 8], haplotype 2221 was protective against an adverse lipid profile that is commonly associated with IR and the metabolic syndrome. Finally, the protective haplotype was associated with lower IMT of the common carotid arteries. This result is consistent with a GWAS showing associations of rs2943634 (being part of the haplotype) with risk of coronary artery disease [6]. While all associations were observed in males, none of the associations reached significance in the smaller population of females.

A new finding of our studies is the association of the protective haplotype with reduced CRP levels. Chronic, subclinical inflammation of adipose tissue significantly contributes to IR [27]. While effects of rs2943641 genotypes on insulin signal transduction in skeletal muscle have been demonstrated, it is not clear whether and how IR in skeletal muscle causes increased CRP levels. Thus, other tissues may contribute to the insulin resistance associated with the polymorphisms studied.

Two recent studies have implicated* IRS1* as the causal locus functionally affected by the polymorphisms. The original study showed moderate associations of rs2943641 with IRS1 protein levels in skeletal muscle in the basal state (*P* = 0.03), but not after* in vivo* stimulation with insulin. Our* IRS1* mRNA results in a smaller population are compatible with the original findings, as subjects homozygous for the less common allele at rs2943641 showed the highest* IRS1* transcript levels. Importantly, the original study showed a strong association of IRS1-phosphatidylinositol-3-OH kinase (PI(3)K) activity with rs2943641 genotypes during* in vivo* insulin infusion (*P* = 0.001). These data strongly suggest genotype effects on insulin signal transduction in skeletal muscle but do not necessarily argue for the causality of* IRS1*, as changes in the amino acid sequence of IRS1 or posttranslational modifications would have to be postulated that affect its association with PI(3)K. However, the three known* IRS1* alleles resulting in amino acid substitutions (rs1801276, rs1801278, and rs12999226) occur with low frequencies (<0.06) [9, 28, 29], while the frequency of the protective rs2943641 allele is much greater (0.334–0.374). In another study, rs2943650, which is in strong LD with rs2943641, displayed no association with BMI, but with body fat mass, as the “at-risk” allele was associated with a 0.16% lower body fat percentage per copy. The effect was stronger in men than in women. Furthermore, in 4997 men, the major allele was associated with an adverse distribution of body fat in which it reduced subcutaneous fat. As no changes were observed in visceral fat, the ratio of visceral to subcutaneous fat was increased in men, but not in women. In addition, the body fat-decreasing allele was associated with reduced* IRS1* transcript levels in the subcutaneous and visceral adipose tissue in both men and women (*N* > 500), but the association appeared to be stronger in men [8]. Our data in a much smaller population displayed no difference in fat mass or distribution between the most common and the protective haplotype. Another haplotype (1111) that revealed no difference in IR in comparison to the reference haplotype showed marginal effects on the VAT/SAT ratio. Most recently, a meta-analysis comprising up to 322154 subjects reported a sex-specific association of rs2176040, also located in the genomic region of* LOC646736* and upstream of* IRS1*, with BMI in 152893 men [30]. Thus, while there is converging evidence for* IRS1* as the causal locus, it is currently not clear whether insulin signaling in skeletal muscle or adipose tissue biology that may result in insulin resistance is primarily affected by the genomic region upstream of* IRS1*.

Our results do not exclude* IRS1* as the causal locus, but they suggest that* LOC646376* may also contribute to IR-related phenotypes. First,* LOC646736* is localized within the LD block of the informative SNPs; second, the same haplotype was protective against all the interrelated phenotypes studied; third,* LOC646736* transcript levels are mainly expressed in skeletal muscle; fourth, the protective haplotype was associated with the greatest abundance of skeletal muscle* LOC646736* transcripts; and fifth, skeletal muscle* LOC646736* mRNA levels were higher in women than in men.

To this end, we show that two different* LOC646736* transcripts are translated in* in vitro* expression systems and also after transfection of various cell lines. Clearly, it will be of utmost importance to determine whether the putative proteins are expressed in human tissues. It is also possible that* LOC646736* transcripts represent lincRNA, implicated in the coordination of coherent protein responses [23, 31]. Hence, lincRNA might also influence insulin signal transduction. Furthermore, other genetic variabilities including miRNAs, copy-number variants, and* NYAP2*, located in a distinct LD block ~400 kb upstream of* LOC646736*, deserve consideration. NYAP2 is mainly expressed in brain, where it mediates the interaction of PI(3)K with the Wiscott-Aldrich Syndrome Protein Family, member 1 complex, thereby regulating neuronal morphogenesis [32]. miR-5702 is located adjacent to* IRS1* and outside of the* LOC646736* LD block. Finally, a known copy-number variant (CNV1152.1) is tagged by three SNPs (rs1849878, rs2673148, and rs2713547, *r* ^2^ ~ 0.5) that are in moderate LD with rs2943634 (*r* ^2^ ~ 0.44) [4]. However, neither the tagging SNPs nor the specific copy-number repeat have shown associations with type 2 diabetes [33].

As insulin signaling and skeletal muscle metabolism are thought to be similar between humans and rodents, the absence of* LOC64736* homologs in rodents may argue against its role in IR. However, additional control mechanisms may have evolved in higher mammalian species. An example for such fine-tuning is* TBC1D3*, a hominoid-specific gene that promotes insulin signaling by delaying its degradation through suppression of phosphorylation of critical serine residues [34].

In conclusion, our studies have defined a haplotype upstream of* IRS1* that protects against IR and related metabolic abnormalities. Our data are consistent with a role of* LOC646736* in IR-related phenotypes. Clearly, functional studies will be required to unravel the complexity associated with this genomic region.

## Supplementary Material

Supplementary Material contains 17 Supplementary Tables showing additional clinical data of study subjects, sequences of primers used for PCR and generation of *in vitro* translation and eGFP constructs, genotypes by SNPs and sex, pairwise linkage disequilibria between SNPs and associations of haplotypes with traits by sex.

## Figures and Tables

**Table 1:** Clinical characteristics of SAPHIR participants without diabetes mellitus by haplotypes.

Trait	Haplotypes						*P* ^(1)^
	1111	1112	1121	1221	2112	2221	
Frequencies	0.169	0.397	0.029	0.014	0.029	0.322	
Age, years	25.7 (25.2–26.1)	26.1 (25.8–26.4)	25.1 (23.8–26.4)	26.1 (24.3–28.0)	25.8 (24.7–26.9)	25.7 (24.4–26.1)	n.s.
BMI, kg/m^2^ ^(2)^	11.9 (10.9–12.8)	11.9 (10.9–12.8)	11.9 (10.7–13.1)	12.0 (10.4–13.5)	11.8 (10.5–13.0)	11.9 (10.9–12.8)	n.s.
Lean body mass, kg^(2)^	45.2 (43.3–47.1)	45.3 (43.5–47.0)	45.0 (42.6–47.4)	46.0 (42.7–49.4)	45.9 (43.4–48.4)	45.2 (43.4–47.0)	n.s.
Fat mass, kg^(2)^	1.81 (−0.21–3.84)	2.01 (0.00–4.01)	2.46 (−0.12–5.05)	0.69 (−2.45–3.83)	1.00 (−1.54–3.54)	2.09 (0.09–4.08)	n.s.
Body fat, %^(2)^	−0.4 (−2.3–1.4)	−0.2 (−2.0–1.6)	0.2 (−2.5–2.8)	−1.9 (−4.8–1.0)	−1.6 (−3.9–0.7)	−0.1 (−1.9–1.7)	n.s.
VAT, cm^2^ ^2^	1.2 (−9.6–12.1)	4.5 (−5.8–14.9)	4.0 (−9.6–17.6)	6.7 (−8.8–22.3)	−0.2 (−14.0–13.5)	5.7 (−4.6–16.1)	n.s.
SAT, cm^2^ ^2^	17.9 (−5.7–41.6)	16.4 (−6.7–29.6)	27.3 (−2.1–56.6)	26.2 (−10.3–62.6)	17.2 (−12.6–47.0)	19.2 (−3.4–41.9)	n.s.
VAT/SAT^(2)^	0.18 (0.13–0.23)^(3)^	0.20 (0.15–0.25)	0.18 (0.11–0.25)	0.19 (0.10–0.28)	0.17 (0.10–0.24)	0.20 (0.15–0.25)	n.s.
Glucose, mg/dL^(5)^	30.6 (27.8–33.4)	30.9 (28.2–33.6)	30.8 (26.8–34.9)	31.9 (26.9–37.1)	29.1 (26.1–32.1)	30.8 (28.1–33.6)	n.s.
Insulin, pmol/L^(5)^	−31 (−38–−24)	−30 (−36–−23)	−31 (−39–−22)	−24 (−32–−16)^(3)^	−30 (−38–−23)	−32 (−39–−26)^(3)^	0.0562
HOMA-IR^(5)^	−1.46 (−1.76–−1.17)	−1.41 (−1.69–−1.13)	−1.45 (−1.80–−1.10)	−1.14 (−1.49–−0.79)^(4)^	−1.45 (−1.77–−1.13)	−1.52 (−1.80–−1.23)^(4)^	0.0293
Cholesterol, mg/dL^(5)^	80 (69–91)	80 (69–91)	74 (61–87)	89 (73–104)	77 (63–91)	79 (68–90)	n.s.
HDL chol, mg/dL^(5)^	36 (32–40)	36 (32–39)	34 (29–39)	37 (30–43)	36 (31–41)	37 (33–41)	n.s.
LDL chol, mg/dL^(5)^	51 (40–61)	50 (40–61)	43 (30–55)	57 (43–71)	45 (32–58)	49 (39–60)	n.s.
Triglycerides, mg/dL^(5)^	−3 (−27–22)	2 (−22–26)	4 (−22–31)	−5 (−48–37)	3 (−28–34)	−4 (−29–21)^(3)^	n.s.
CRP, mg/L^(5)^	−2.58 (−3.29–−1.88)^(3)^	−2.39 (−3.05–−1.72)	−2.40 (−3.27–−1.52)	−2.40 (−3.46–−1.34)	−2.51 (−3.39–−1.63)	−2.56 (−3.24–−1.87)^(4)^	n.s.

Results represent expected means (95% CI) of untransformed data; for haplotype designation, 1 or 2 refers to the major or minor alleles, respectively, in the following order: rs7578326, rs2943634, rs2943641, and rs2713538. ^(1)^ *P* values for global haplotype effect, ^(2)^adjusted for age and sex, ^(3)^ *P* < 0.05 and ^(4)^ *P* < 0.01, relative to the most frequent haplotype, ^(5)^adjusted for age, sex, and BMI, *N* = 1582, *N* = 1500 for lipids (subjects on lipid lowering drugs excluded). *N* = 1521 for body composition, *N* = 1437 for VAT and SAT, and *N* = 1563 for CRP (subjects with values >20 mg/L excluded); n.s., not significant; VAT, visceral adipose tissue; SAT, subcutaneous adipose tissue.

**Table 2:** Haplotypes and associated risk for type 2 diabetes mellitus.

Haplotype	Frequencies (%)		Odds ratio (95% CI)	
	Controls (*N* = 1350)	Cases (*N* = 600)	Univariate analysis	Multivariate analysis^1^
1111	0.164	0.164	0.90 (0.73–1.10)	0.89 (0.72–1.10)
1112	0.395	0.441	1.00	1.00
1121	0.030	0.032	0.94 (0.62–1.41)	0.90 (0.58–1.38)
1221	0.014	0.011	0.71 (0.37–1.37)	0.82 (0.42–1.61)
2112	0.029	0.026	0.80 (0.51–1.24)	0.82 (0.51–1.29)
2221	0.329	0.288	0.79 (0.67–0.93)^2^	0.78 (0.66–0.92)^3^

For haplotype designation, 1 or 2 refers to the major or minor alleles, respectively, in the following order: rs7578326, rs2943634, rs2943641, and rs2713538; global haplotype effects for univariate and multivariate analyses *P* < 0.05; ^1^adjusted for age, sex, and BMI; ^2^ *P* = 0.0051; ^3^ *P* = 0.0035.

**Table 3:** Haplotypes and intima media thickness of common carotid arteries.

Haplotype	Frequencies (%)	IMT (mm)	*P*
1111	0.166	0.097 (0.060–0.135)	n.s.
1112	0.402	0.103 (0.065–0.141)	Reference
1121	0.027	0.111 (0.069–0.153)	n.s.
1221	0.013	0.101 (0.044–0.158)	n.s.
2112	0.027	0.105 (0.063–0.147)	n.s.
2221	0.322	0.092 (0.056–0.129)	0.0265

For haplotype designation, 1 or 2 refers to the major or minor alleles, respectively, in the following order: rs7578326, rs2943634, rs2943641, and rs2713538. *N* = 1542 (943 men, 599 women); data are expected as means (95% CI) of average IMTs of common carotid arteries, adjusted for age, sex, BMI, hypertension status, smoking status, use of lipid lowering drugs, LDL cholesterol, and CRP. Global haplotype effect n.s.; IMT, intima media thickness; n.s., not significant.

**Table 4:** Haplotypes and *LOC646736* and *IRS1* transcript levels in skeletal muscle.

Haplotype	Frequencies (%)	*LOC646736 *		*IRS1 *	
		Log transcript level (AU)	*P*	Log transcript level (AU)	*P*
1111	0.164	0.258 (−0.006–0.522)	n.s.	0.574 (0.327–0.821)	n.s.
1112	0.411	0.257 (−0.006–0.522)	Reference	0.534 (0.345–0.725)	Reference
1121	0.022	0.170 (−0.385–0.726)	n.s.	0.434 (−0.240–1.109)	n.s.
1221	0.017	0.221 (−1.747–2.189)	n.s.	0.480 (−1.283–2.244)	n.s.
2112	0.028	0.484 (0.005–0.969)	n.s.	0.438 (−0.012–0.889)	n.s.
2221	0.336	0.418 (0.113–0.724)	0.0096	0.625 (0.369–0.881)	0.1928

For haplotype designation, 1 or 2 refers to the major or minor alleles, respectively, in the following order: rs7578326, rs2943634, rs2943641, and rs2713538; data are means (95% CI) in arbitrary units (AU) of 92 subjects, adjusted for age, sex, and BMI; global haplotype effect *P* = 0.0408 for *LOC646736* and *P* = n.s.; n.s., not significant. Transcript quantification of *LOC646736* (with primers targeting exons 6 and 7) and *IRS1* are normalized for *RPL0*.
