# Supplementary material for: Associations of Haplotypes Upstream of IRS1 with Insulin Resistance, Type 2 Diabetes, Dyslipidemia, Preclinical Atherosclerosis, and Skeletal Muscle LOC646736 mRNA Levels
Source: J Diabetes Res. 2015 May 18;2015:405371. doi: 10.1155/2015/405371 (PMC4451528; doi:10.1155/2015/405371)
Supplement: Supplementary file 1 — Supplementary Material contains 17 Supplementary Tables showing additional clinical data of study subjects, sequences of primers used for PCR and generation of in vitro translation and eGFP constructs, genotypes by SNPs and sex, pairwise linkage disequilibria between SNPs and associations of haplotypes with traits by sex. [file 405371.f1.pdf]

ELECTRONIC SUPPLEMENTARY MATERIAL

Supplementary Tables

Supplementary Table 1 Prevalence of obesity, glucose intolerance and type 2 diabetes in SAPHIR participants by sex

Gluose metabolism	Men		Women	
	BMI<30 kg/m^2	BMI≥30 kg/m^2	BMI <30 kg/m^2	BMI≥30 kg/m^2
Glucose tolerant	713 (69.4)	121 (11.8)	428 (68.8)	88 (14.1)
Glucose intolerant	113 (11.0)	36 (3.5)	58 (9.3)	25 (4.0)
Diabetes	25 (2.4)	20 (1.9)	8 (1.3)	15 (2.4)

Results are for 1028 male and 622 female participants; percentages of all men or women in parentheses.

Supplementary Table 2 Clinical characteristics of male and female SAPHIR participants without type 2 diabetes

Trait	Men	Women	*p*
N	983	599	
Age, years	49.1 (5.5)	56.0 (4.4)	n.d.
BMI, kg/m^2 [1]	26.8 (3.6)	26.3 (4.6)	0.0161
BMI≥30 kg/m^2	157 [16]	113 [18.9]	n.s.
Lean body mass, kg [1]	67.2 (9.2)	48.4 (5.9)	<0.001
Fat mass, kg [1]	17.3 (7.9)	21.8 (9.4)	<0.001
Body fat, % [1]	20.1 (8.0)	29.9 (7.9)	<0.001
VAT, cm^2 [1]	85.5 (44.7)	63.4 (42.0)	<0.001
SAT, cm^2 [1]	174.1 (84.0)	243.2 (113.4)	<0.001
VAT/SAT [1]	0.521 (0.230)	0.282 (0.196)	<0.001
Glucose, mg/dl [2]	92.5 (9.3)	89.0 (9.2)	<0.001
Insulin, pmol/l [2]	7.25 (4.8)	6.81 (3.6)	0.0560
HOMA-IR[5]	1.69 (1.21)	1.53 (0.91)	0.0044
Cholesterol, mg/dl [2,3]	230 (39)	232 (40)	n.s.
HDL chol, mg/dl [2,3]	56 (13)	67 (16)	<0.001
LDL chol, mg/dl [2,3]	149 (36)	144 (37)	0.016
Triglycerides, mg/dl [2,3]	131 (97)	104 (54)	<0.001
CRP, mg/l [2,4]	2.01 (2.33)	2.87 (2.86)	0.001

Results are numbers of observations [percentages] or average values (SD); [1]adjusted for age, [2]adjusted for age and BMI; [3]30 male and 52 female on lipid lowering drugs excluded. [4]11 males and 8 females with CRP-values >20 mg/l excluded; body composition was available in 941 male and 580 females; VAT and SAT was available in 897 male and 540 female subjects; VAT, visceral adipose tissue; SAT, subcutaneous adipose tissue; n.d., not determined.

Supplementary Table 3 Obesity in cases with type 2 diabetes and glucose tolerant controls by sex

Trait	Diabetes		Controls		p^1	p^2	p^3
	Men (N=338)	Women (N=262)	Men (N=834)	Women (N=516)			
BMI≥30 kg/m^2	117 [34.6]	107 [40.8]	121 [14.5]	88 [17.1]	<0.001	0.0477	n.s.

Results are numbers of observation [percentages]; [1,2,3] p-values for BMI>30 kg/m^2, sex and interaction between sex and BMI>30 kg/m^2 in logistic regression with diabetes/control as dependent variable.

Supplementary Table 4 Clinical characteristics of SAPHIR participants with IMT measurements

Trait	Men	Women	*p*
N	943	599	
Age, years	49.3 (5.4)	56.1 (4.3)	n.d.
BMI, kg/m^2 [1]	27.3 (3.7)	26.1 (4.7)	<0.001
BMI≥30 kg/m^2	169 [17.9]	122 (20.4)	n.s.
type 2 diabetes, n	40 [4.2]	22 [3.7]	n.s.
Hypertension, n	371 (39.3)	239 (39.9)	n.s.
Glucose intolerance, n	143 [15.2]	83 [13.9]	n.s.
Lipid lowering drugs, n	27 [2.9]	46 [7.7]	<0.001
Current smoking, n	217 [23.0]	77 [12.9]	0.0000
Cholesterol, mg/dl [2]	229 (40)	231 (40)	n.s.
HDL cholesterol, mg/dl [2]	56 (13)	67 (17)	<0.001
LDL cholesterol, mg/dl [2]	148 (36)	144 (37)	0.0413
Triglycerides, mg/dl [2]	136 (101)	107 (55)	<0.001
CRP, mg/l [2]	2.51 (7.40)	3.48 (4.83)	<0.001
IMT carotid arteries, mm	0.760 (0.124)	0.768 (0.131)	n.s.
IMT carotid arteries, mm [2]	0.794 (0.124)	0.735 (0.131)	<0.001

Results are numbers of observations [percentages] or average values (SD); [1]adjusted for age, [2]adjusted for age and BMI; IMT, intima-media thickness of carotid arteries; n.d., not determined.

Supplementary Table 5 Primers for SYBR Green real-time PCR

LOC646736 exon 1 forward	5´ AATGAACAAGTCCACCCCAGCG 3´
LOC646736 exon 6 foward	5´ ATGCCTTGGGAGAGAGCTGTTA 3´
LOC646736 exon 7 reverse	5´ GAGGTTCATGAATTTGCTACTCA 3´
IRS1 forward	5´ CCACTCGGAAAACTTCTTCTTCAT
IRS1 reverse	5´ AGAGTCATCCACCTGCATCCA

Supplementary Table 6 PCR primers for in vitro translation constructs (pT7CFE1-CHis expression vector)

LOC646736_Ex1-3_CFE forward	5´ GAGAggatccATGTCAGTGTTGAAGGTTAC 3´<br>BamHI
LOC646736_Ex1-3_CFE reverse	5´ GAGActcgagGGCTTGATTAATTTCTTCCTC 3´<br>XhoI
LOC646736_Ex5-7_CFE forward	5´ GAGAggatccAATATGGAATGCTCACTCC 3´<br>BamH1
LOC646736_Ex5-7_ CFE reverse	5´ GAGActcgagCATCTTCTCCCTTCTCAG 3´<br>XhoI

Supplementary Table 7 PCR primers for eGFP constructs (pEGFP-N1 expression vector)

LOC646736_Ex1-3_GFP forward	5´ GAGActcgagATGTCAGTGTTGAAGGTTAC 3´<br>XhoI
LOC646736_Ex1-3_GFP reverse	5´ GAGAggatccGGCTTGATTAATTTCTTCCTC 3´<br>BamH1
LOC646736_Ex5-7_GFP forward	5´ GAGActcgagAATATGGAATGCTCACTCC 3´<br>XhoI
LOC646736_Ex5-7_GFP reverse	5´ GAGAggatccCATCTTCTCCCTTCTCAG 3´<br>BamHI

Supplementary Table 8 Clinical characteristics of subjects with skeletal muscle biopsy

Trait	BMI>30 kg/m^2	BMI<30 kg/m^2	*p*
N, m/f	23/50	9/10	n.s.
Age, years	38.2 (11.1)	46.5 (13.6)	0.0065
BMI, kg/m^2 [1]	44.3 (8.1)	24.8 (3.0)	n.d.
Glucose, mg/dl [2]	91.3 (12.1)	88.6 (8.4)	n.s.
Insulin, pmol/l [2]	14.9 (13.6)	5.3 (3.6)	0.0039
HOMA-IR[2]	3.43 (3.14)	1.18 (0.90)	0.0031
Cholesterol, mg/dl [2]	191 (36)	186 (46)	n.s.
HDL chol, mg/dl [2]	43 (17)	49 (12)	n.s.
LDL chol, mg/dl [2]	113 (34)	111 (42)	n.s.
Triglycerides, mg/dl [2]	257 (364)	197 (191)	n.s.
CRP, mg/l [2]	7.15 (6.39)	1.14 (0.91)	<0.001
LOC646736 mRNA, AU	6.52 (5.84)	7.11 (5.42)	n.s.
IRS1 mRNA, AU	14.9 (19.3)	14.5 (17.4)	n.s.

Results are means (SD); AU, arbitrary units; [1]adjusted for age; [2]adjusted for age and sex; n.d., not determined.

Supplementary Table 9 Genotypes in SAPHIR population

SNP	Sex	Wild-type	Heterozygous	Variant	MAF	p^a
rs7578326	All	658 (0.398)	752 (0.456)	240 (0.146)	0.373	0.2732
	Male	417 (0.406)	454 (0.442)	157 (0.153)	0.374	0.0633
	Female	241 (0.388)	298 (0.479)	83 (0.133)	0.373	0.05446
rs2943634	All	642 (0.389)	768 (0.466)	240 (0.146)	0.378	0.6452
	Male	401 (0.390)	468 (0.455)	159 (0.155)	0.382	0.2279
	Female	241 (0.388)	300 (0.482)	81 (0.130)	0.371	0.4108
rs2943641	All	681 (0.413)	748 (0.453)	221 (0.134)	0.357	0.4650
	Male	430 (0.418)	453 (0.441)	145 (0.141)	0.361	0.1322
	Female	251 (0.404)	295 (0.474)	76 (0.122)	0.359	0.4529
rs2713538	All	477 (0.289)	829 (0.502)	344 (0.209)	0.460	0.6578
	Male	307 (0.299)	497 (0.484)	224 (0.218)	0.460	0.3771
	Female	170 (0.273)	332 (0.534)	120 (0.193)	0.460	0.0633

Numbers represent counts (frequencies); MAF, minor allele frequency; [a]deviation from Hardy-Weinberg equilibrium.

Supplementary Table 10 Standardized pairwise linkage disequilibrium coefficients (D´) and r^2 values of SNPs

	rs7578326	rs2943634	rs2943641	rs2713538
rs7578326	-	0.92	0.80	-0.75
rs2943634	0.796	-	0.89	-0.89
rs2943641	0.617	0.737	-	-0.92
rs2713538	0.293	0.384	0.431	-

SNPs were determined in 1650 participants of the SAPHIR study. D´, upper right and r^2 lower left.

Supplementary Table 11 Clinical characteristics of male SAPHIR participants without diabetes mellitus by haplotypes

Trait	Haplotypes						p^a
	1111	1112	1121	1221	2112	2221	
Frequencies	0.167	0.394	0.029	0.015	0.030	0.325	
Age (years)	24.4 (23.9-24.9)	24.8 (24.5-25.1)	23.8 (22.4-25.3)	24.9 (22.6-27.2)	24.1 (22.7-25.5)	24.4 (24.1-24.8)	NS
BMI (kg/m^2)	10.7 (9.6-11.8)	10.9 (9.9-12.0)	10.6 (9.2-12.1)	11.8 (10.2-13.4)	11.1 (9.8-12.5)	10.9 (9.9-12.0)	NS
Lean body mass (kg)	35.2 (32.3-38.1)	35.1 (32.4-37.9)	34.2 (30.5-38.0)	36.9 (32.2-41.6)	36.5 (32.9-40.2)	34.8 (32.1-37.6)	NS
Fat mass (kg)	3.0 (0.6-5.4)	3.4 (1.0-5.8)	3.4 (-0.2-7.0)	3.6 (0.2-7.0)	2.5 (-0.3-5.4)	3.6 (1.3-6.0)	NS
Body fat (%)	4.7 (1.5-6.4)	4.4 (2.1-6.9)	4.9 (1.3-8.5)	3.7 (0.1-7.3)	2.9 (0.1-5.8)	4.8 (2.4-7.2)	NS
VAT (cm^2)	-14.8(-29.0- -0.49)	-9.6 (-23.3-4.2)	-10.3 (-28.4-7.89)	2.7 (-16.6-22.1)	-15.1 (-32.6-2.37)	-9.6 (-23.2-3.87)	NS
SAT (cm^2)	59.5 (32.3-86.7)	61.25 (34.7-87.8)	60.3 (24.9-95.7)	78.9 (40.8-117.1)	67.2 (34.6-99.7)	65.9 (40.6-91.3)	NS
VAT/SAT	0.01 (-0.06-008)[b]	0.04 (-0.03-0.11)	0.04 (-0.05-0.13)	007 (-0.05-0.18)	0.01 (-0.10- 0.10)	0.04 (-0.03-0.11)	NS
Glucose (mg/dl)	36.4 (33.6-39.3)	36.6 (33.7-39.5)	35.9 (31.6-40.2)	37.9 (32.4-43.4)	35.6 (32.0-39.1)	36.5 (33.8-39.3)	NS
Insulin (pmol/l)	-45 (-54- -36)	-44 (-53- -35)	-44 (-55- -33)	-37(-48- -25)[c]	-46 (-56- -35)	-46 (-55- -38)	NS
HOMA-IR	-2.08 (-2.48- -1.68)	-2.03 (-2.43- -1.64)	-2.05 (-2.53- -1.56)	-1.69 (-2.17- -1.21)[d]	-2.09 (-2.54- -1.65)	-2.13 (-2.52- -1.74)[b]	NS
Cholesterol (mg/dl)	73 (59-87)	73 (59-87)	68 (51-84)	81 (60-102)	68 (50-85)	71 (57-85)	NS
HDL chol (mg/dl)	42 (37-47)	41 (36-46)	40 (33-46)	44 (35-53)	42 (36-48)	43 (38-47)[b]	NS
LDL chol (mg/dl)	46 (33-59)	46 (33-59)	36 (22-51)[b]	52 (34-71)	37 (21-53)	44 (31-57)	NS
Triglycerides (mg/dl)	-45 (-82- -8)	-39 (-75- -2)	-30 (-69-10)	-63 (-145-18)	-32 (-77-13)	-46 (-84- -8)	NS
CRP (mg/l)	-1.51 (-2.44- -0.57)[c]	-1.24 (-2.10- -0.37)	-1.03 (-2.09- 0.04)	-0.88 (-2.12- 0.37)	-1.59 (-2.67- -0.53)	-1.48 (-2.37- -0.58) [b]	0.0164

Results represent expected means (95% CI) of untransformed data; for haplotype designation, 1 or 2 refers to the major or minor alleles, respectively, in the following order: rs7578326, rs2943634, rs2943641, rs2713538;[a]*p*-values for global haplotype effect; N= 983, N=953 for lipids (subjects using lipid lowering drugs were excluded), N=941 for body composition, N=897 for VAT and SAT and N= 972 for CRP (subjects with values >20 mg/l excluded); [b]$P<0.05$, [c]$P<0.02$, [d]$P<0.01$; body composition or metabolic variables were adjusted for age or age and BMI, respectively; VAT, visceral adipose tissue; SAT, subcutaneous adipose tissue; NS, not significant.

Supplementary Table 12 . Clinical characteristics of female SAPHIR participants without diabetes mellitus by haplotypes

Trait	Haplotypes						p^a
	1111	1112	1121	1221	2112	2221	
Frequencies	0.171	0.402	0.031	0.013	0.032	0.325	
Age (years)	27.6 (26.9-28.29)	28.1 (27.8-28.5)	27.5 (25.7-29.3)	28.9 (26.9-30.9)	28.8 (27.5-30.1)	28.0 (27.6-28.4)	NS
BMI (kg/m^2)	12.1 (9.7-14.5)	11.9 (9.5-14.3)	12.2 (9.6-14.7)	10.5 (5.9-15.0)	11.3 (8.3-14.3)	11.8 (9.4-14.2)	NS
Lean body mass (kg)	28.3 (25.8-30.9)	28.6 (26.1-31.1)	29.1 (26.3-32.0)	27.7 (21.7-33.8)	28.0 (24.3-31.7)	29.1 (26.6-31.5)	NS
Fat mass (kg)	9.0 (4.0-14.0)	8.8 (3.7-13.9)	9.8 (4.5-15.1)	5.1 (-3.3-13.5)	7.8 (1.5-14.0)	8.6 (3.6-13.6)	NS
Body fat (%)	10.3 (6.3-14.4)	10.2 (6.1-14.2)	10.5 (5.7-15.2)	7.0 (0.9-13.1)	9.0 4.0-14.0)	9.7 (5.7-13.8)	NS
VAT (cm^2)	-8.1 (-32.0-15.85)	-8.4 (-32.2-15.5)	-9.6 (-35.8-16.6)	-24.2 (-65.6-17.2)	-11.0 (-40.4-18.4)	-4.3 (-28.3-19.6)	NS
SAT (cm^2)	60.4 (-1.7-122.4)	52.4 (-9.4-114.3)	83.2 (16.9-149.6)	50.4 (43.2-143.9)	44.3 (-30.1-118.7)	51.8 (-10.9-114.3)	NS
VAT/SAT	0.07 (-0.05-0.18)	0.09 (-0.04-0.21)	0.03 (-0.14-0.20)	0.01 (-0.25-0.26)	0.08 (-0.06- 0.22)	0.09 (-0.02-0.21)	NS
Glucose (mg/dl)	27.3 (19.9-34.7)	28.1 (20.9-35.3)	28.3 (18.5-38.0)	29.7 (18.7-40.6)	25.0 (18.9-31.1)	28.2 (20.1-36.3)	NS
Insulin (pmol/l)	-16 (-37- -6)	-15 (-25- -4)	-16 (-28- -3)	-16(-31- -0)	-15 (-27- -3)	-17 (-27- -7)	NS
HOMA-IR	-0.84 (-1.31- -0.37)	-0.80 (-1.26- -0.33)	-0.86 (-1.40- -0.32)	-79 (-1.44- -0.14)	-0.83 (-1.38- -0.28)	-0.90 (-1.37- -0.44)	NS
Cholesterol (mg/dl)	92 (67-117)	91 (66-116)	85 (56-114)	95 (65-124)	91 (58-123)	91 (65-117)	NS
HDL chol (mg/dl)	44 (35-54)	45 (36-55)	43 (33-54)	44 (32-55)	45 (33-57)	45 (36-55)	NS
LDL chol (mg/dl)	50 (27-74)	48 (24-71)	44 (15-73)	49 (23-76)	49 (18-79)	49 (25-72)	NS
Triglycerides (mg/dl)	-1 (-32-30)	0 (-32- 32)	-6 (-43-30)	12 (-26-52)	-14 (-59-31)	-4 (-36-29)	NS
CRP mg/l)	-2.59 (-4.31- -0.88)	-2.51 (-4.27- -0.76)	-2.93 (-5.02- -0.84)	-3.10 (-6.69- 0.50)	-2.18 (-4.18- -0.18)	-2.54 (-4.27- -0.81)	NS

Results represent expected means (95% CI) of untransformed data; for haplotype designation, 1 or 2 refers to the major or minor alleles, respectively, in the following order: rs7578326, rs2943634, rs2943641, rs2713538; [a]*p*-values for global haplotype effect; N= 599, N=547 for lipids (subjects using lipid lowering drugs were excluded), N=580 for body composition, N=540 for VAT and SAT and N=591 for CRP (subjects with values>20 mg/L excluded); body composition or metabolic variables were adjusted for age or age and BMI, respectively; VAT, visceral adipose tissue; SAT, subcutaneous adipose tissue; NS, not signigicant.

Supplementary Table 13 Polymorphisms and associated risk of type 2 diabetes mellitus

		Cases		Controls			Odds Ratio (95% CI)	
SNP	Genotype	N	%	N	%	p^a	Univariate Analysis	Multivariate Analysis[b]
rs7578326	AA	252	0.4203	518	0.384		1.00	1.00
	AG	282	0.470	624	0.462		0.93 (0.76-1.14)	0.93 (0.75-1.16)
	GG	66	0.110	208	0.154	0.02758	0.65 (0.48-0.89)	0.61 (0.44-0.85)
rs2943634	CC	265	0.442	544	0.403		1.00	1.00
	CA	275	0.458	615	0.456		0.92 (0.75-1.13)	0.94 (0.75-1.16)
	AA	60	0.100	191	0.142	0.0293	0.64 (0.47-0.89)	0.60 (0.42-0.84)
rs2943641	CC	251	0.418	509	0.377		1.00	1.00
	CT	283	0.472	631	0.467		0.91 (0.74-1.12)	0.92 (0.74-1.14)
	TT	66	0.110	210	0.156	0.0190	0.64 (0.47-0.87)	0.59 (0.42-0.84)
rs2713538	AA	152	0.253	398	0.295		1.00	1.00
	AG	307	0.512	687	0.509		1.17 (0.92-1.47)	1.24 (0.97-1.59)
	GG	141	0.235	265	0.196	0.0630	1.39 (1.05-1.84)	1.48 (1.10-1.88)

[a]χ^2 analysis; [b]logistic regression analysis adjusted for age, sex and BMI.

Supplementary Table 14 Haplotypes and associated risk of type 2 diabetes mellitus in men

Haplotype	Frequencies (%)		Odds Ratio (95% CI)	
	Controls (N=834)	Cases (N=338)	Univariate Analysis	Multivariate Analysis[a]
1111	0.166	0.162	0.85 (0.66-1.10)	0.84 (0.64-1.10)
1112	0.395	0.453	1.00	1.00
1121	0.030	0.028	0.81 (0.45-1.45)	0.82 (0.45-1.50)
1221	0.015	0.011	0.60 (0.25-1.44)	0.71 (0.30-1.67)
2112	0.030	0.025	0.73 (0.41-1.32)	0.80 (0.44-1.44)
2221	0.325	0.274	0.74 (0.59-0.92)[b]	0.76 (0.60-0.95)[c]

For haplotype designation, 1 or 2 refers to the major or minor alleles, respectively, in the following order: rs7578326, rs2943634,rs2943641, rs2713538; global haplotype effects for univariate analysis *p*= 0.04854; [a]adjusted for age and BMI; [b]*p*=0.0057, [c]*p*=0.0183.

Supplementary Table 15 Haplotypes and associated risk of type 2 diabetes mellitus in women

Haplotype	Frequencies (%)		Odds Ratio (95% CI)	
	Controls (N=516)	Cases (N=262)	Univariate Analysis	Multivariate Analysis[a]
1111	0.163	0.166	0.96 (0.70-1.32)	0.98 (0.69-1.38)
1112	0.393	0.425	1.00	1.00
1121	0.031	0.037	1.10 (0.61-2.01)	1.13 (0.62-2.08)
1221	0.012	0.012	0.93 (0.33-2.58)	1.09 (0.31-3.84)
2112	0.028	0.026	0.88 (0.44-1.76)	0.98 (0.47-2.03)
2221	0.333	0.306	0.87 (0.68-1.12)	0.81 (0.62-1.06)[b]

For haplotype designation, 1 or 2 refers to the major or minor alleles, respectively, in the following order: rs7578326, rs2943634, rs2943641, rs2713538; global haplotype effects for univariate and multivariate analyses not significant; [a]adjusted for age and BMI;
[b]*p*=0.1354.

Supplementary Table 16 Haplotypes and intima-media thickness of common carotid arteries in men

Haplotype	Frequencies (%)	IMT (mm)	*p*
1111	0.164	0.046 (0.001-0.090)	n.s.
1112	0.400	0.047 (0.001-0.094)	reference
1121	0.028	0.049 (0.001-0.102)	n.s.
1221	0.013	0.023 (0.051-0.097)	n.s.
2112	0.029	0.076 (0.023-0.128)	n.s.
2221	0.324	0.036 (-0.010-0.083)	0.0427

For haplotype designation, 1 or 2 refers to the major or minor alleles, respectively, in the following order: rs7578326, rs2943634, rs2943641, rs2713538; N=943; data are expected means (SD) of average IMTs of common carotid arteries, adjusted for age, BMI, hypertension status, smoking status, use of lipid lowering drugs, LDL cholesterol and CRP; global haplotype effect n.s.; IMT, intima-media thickness; NS, not significant.

Supplementary Table 17. Haplotypes and intima-media thickness of common carotid arteries in women

Haplotype	Frequencies (%)	IMT (mm)	*p*
1111	0.171	0.101 (0.022-0.179)	n.s.
1112	0.403	0.115 (0.036-0.194)	reference
1121	0.028	0.131 (0.044-0.218)	n.s.
1221	0.015	0.149 (0.039-0.259)	n.s.
2112	0.026	0.074 (-0.015-0.163)	n.s.
2221	0.317	0.103 (0.025-0.181)	n.s.

For haplotype designation, 1 or 2 refers to the major or minor alleles, respectively, in the following order: rs7578326, rs2943634, rs2943641, rs2713538; N=599; data are expected means (SD) of average IMTs of common carotid arteries, adjusted for age, BMI, hypertension status, smoking status, menopausal status, use of lipid lowering drugs, LDL cholesterol and CRP; global haplotype effect NS; IMT, intima-media thickness; NS, not significant.
